# Coronary artery aneurysm and early calcification in Kawasaki disease with *METTL23* mutation

**DOI:** 10.1093/ehjcr/ytaf195

**Published:** 2025-04-15

**Authors:** Takahiro Maeda, Sho Imai, Tomohiro Nawa

**Affiliations:** Department of Pediatric Cardiology, Hokkaido Medical Center for Child Health and Rehabilitation, 1-1-240-6, Kanayama, Teine-ku, Sapporo, Hokkaido 006-0041, Japan; Radiology Department, Hokkaido Medical Center for Child Health and Rehabilitation, 1-1-240-6, Kanayama, Teine-ku, Sapporo, Hokkaido 006-0041, Japan; Department of Pediatric Cardiology, Hokkaido Medical Center for Child Health and Rehabilitation, 1-1-240-6, Kanayama, Teine-ku, Sapporo, Hokkaido 006-0041, Japan

**Keywords:** Kawasaki disease, Coronary aneurysm, Coronary artery bypass grafting

A 1-year and 3-month-old boy with intellectual developmental delay presented with fever for 2 days. The clinical findings included conjunctival erythema, strawberry tongue, non-suppurative lymphadenopathy, and an atypical skin rash, leading to a diagnosis of Kawasaki disease. The patient received intravenous high-dose immunoglobulin therapy (2 g/kg/dose) on Days 2 and 4 of fever but was intravenous immunoglobulin therapy (IVIG)-resistant. Additionally, he received methylprednisolone pulse therapy on Day 6, IVIG on Days 10 and 17, and infliximab on Day 18. On Day 15, giant coronary artery aneurysms measuring 11 mm in Segment 1 and 12 mm in Segment 6, were identified (*Figure 1A*). The patient was treated with warfarin, aspirin, and angiotensin receptor blockers and no clinical events occurred. However, computed tomography (CT) and coronary angiography performed at 3 years and 3 months (*Figure 1B* and *C*, respectively) revealed calcification in the left anterior descending artery. Follow-up echocardiography at 4 years and 2 months suggested progressive thrombus formation and calcification, prompting repeat CT imaging at 4 years and 3 months. The calcification had advanced, and coronary angiography revealed 99% stenosis in Segment 6 (*Figure 1D*). Electrocardiographic changes were observed during the tests. Severe stenotic lesions and clinical signs of ischaemia were observed, indicating the need for coronary artery bypass grafting. Therefore, a left internal thoracic artery to left anterior descending artery (LITA-LAD) bypass was performed at 4 years and 5 months. The patient was discharged on post-operative Day 7, and the graft remained patent with a favourable clinical course. Genetic testing for developmental delay identified a METTL23 mutation that has been associated with intellectual disabilities, leading to the diagnosis of autosomal recessive intellectual developmental disorder.

**Figure 1:**
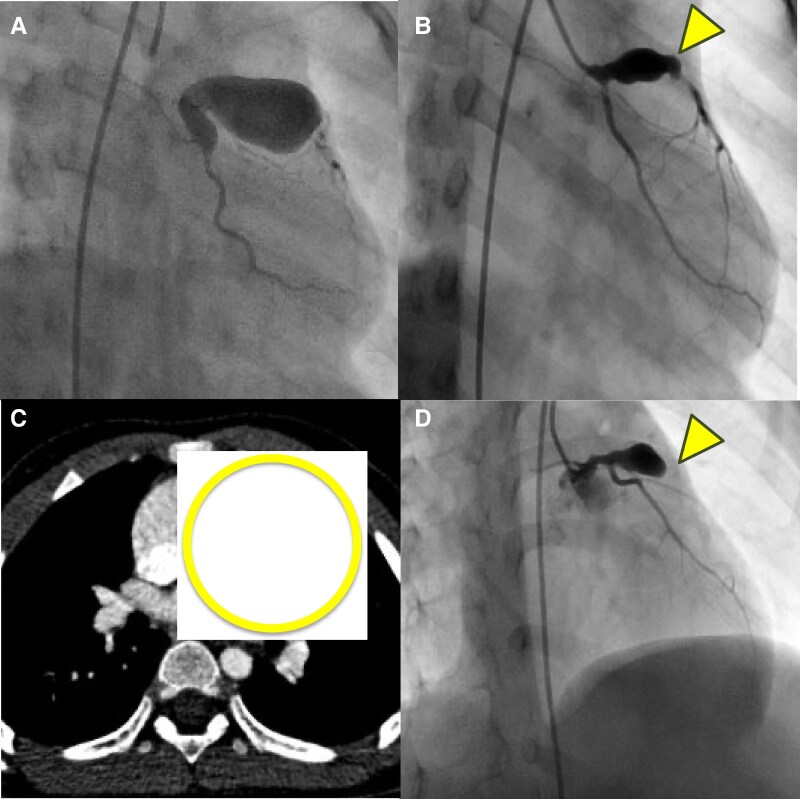
Course of coronary artery aneurysm in this case. Giant coronary aneurysm in Segment 1 and 6 (*A*). Coronary aneurysm in Segment 6 two years after onset (*B, C*). Coronary aneurysm in Segment 6 three years after onset (*D*).

According to Yang *et al*.,^1^ the *METTL23* mutation affects histone H3R17 methylation and is linked to NF-κB-mediated Tumor Necrosis Factorα and IL1B transcription. To date, there have been no reports of Kawasaki disease in patients with *METTL23* mutations. The impact of this mutation on Kawasaki disease remains unclear, though its role in this patient’s refractory course, sequelae, and early calcification may be significant.

##  

**Consent:** This case report was written with patient consent and is in accordance with COPE guidelines.

**Funding:** None declared.

## Data Availability

The data underlying this article will be shared on reasonable request to the corresponding author.
